# Development and Evaluation of the Quality of Life Scale for Children With Visual Impairments in China

**DOI:** 10.3389/fped.2022.739296

**Published:** 2022-03-21

**Authors:** Jie Liu, Richard Evans, Yanjun Wang, Beibei Hu, Yan Tong, Shaoqiong Li, Zhiqiang Tian, Jing Li, Cuihua Zhang, Lu He, Jianzhong Zheng

**Affiliations:** ^1^School of Public Health, Shanxi Medical University, Taiyuan, China; ^2^College of Engineering, Design and Physical Sciences, Brunel University London, London, United Kingdom; ^3^Service Center of Shanxi Medical and Health Institutions, Taiyuan, China; ^4^Center for Information, Chinese Center for Disease Control and Prevention, Beijing, China; ^5^Physiatry Department, Hospital of Traditional Chinese Medicine, Taiyuan, China

**Keywords:** children, reliability, validity, quality of life, physical wellbeing, visual impairments

## Abstract

**Background:**

Visual impairments related to non-correctable vision loss, including blindness and low vision, have been consistently shown to lower a person's health-related quality of life. This study assessed the reliability, validity, and discrimination of the Quality of Life Scale for Children with Visual Impairments (QOLS-CVI) in China.

**Methods:**

The Pediatric Quality of Life Inventory™ 4.0 and World Health Organization Quality of Life-Disability Scale for physical disability were selected to define conceptual frameworks and item libraries based on relevant existing studies. According to two rounds of expert consultations and group discussions, some items were modified, and the draft scale was developed. Two item selection processes based on classical test theory and item response theory were used to conduct a preliminary survey and a formal survey in special schools in Shanxi and Hebei Provinces. Finally, the reliability and validity of the quality of life scale for visually impaired children in China were verified.

**Results:**

The final QOLS-CVI consisted of 38 items, 10 subdomains, and 6 domains. Reliability was verified by Cronbach's alpha coefficient, split-half reliability, and test-retest reliability (Cronbach's alpha for the full scale, 0.841; split-half reliability, 0.629; and test–retest reliability, 0.888). The validity results showed that the multidimensional scale met expectations: exploratory factor analysis and confirmatory factor analysis indicated good fitting models for children with visual impairments.

**Conclusions:**

The QOLS-CVI was determined to be reliable and valid and to have strong feasibility and effectiveness. This scale can be used as an evaluation tool to study the QOL and social-participation ability of children with visual impairments.

## Introduction

In the Sixth National Population Census, conducted in November 2010 by the National Bureau of Statistics of the People's Republic of China, the number of citizens with disabilities was ~85.02 million, representing 6.34% of China's 1.33 billion population. Of these, individuals with visual impairments accounted for 12.63 million (14.86%), with 494,000 of these being children aged 0–14 years (1).

Presently, for visually impaired children in China, clinical medicine mainly focuses on diagnosing the degree and cause of visual disability, and great achievements have been made, enabling appropriate remedy of the defects of visually impaired children with the help of medical equipment. However, studies on the special psychological and cognitive development characteristics, social communication ability, and early rehabilitation assessment of visually impaired children are still few (2). In terms of schooling, it is difficult to find suitable schools for the blind and most of them are enrolled in regular school classes. Although the government is paying more attention to visually impaired children, for example, the government proposed an early education and rehabilitation intervention for children in the “Expanded Core Curriculum (ECC)” as early as possible, and put forward the concept of integrated education and relevant rehabilitation countermeasures, among others. However, several problems exist regarding the quality of life of visually impaired children (3). For example, the concept of ECC occurring in ordinary schools is worse than when it occurs in the blind schools. However, there is a gap in the entire educational teaching level in the blind schools (4). Hence, there are still several problems to be addressed. In terms of employment opportunities, the visually impaired face huge challenges, including low employment rate, large gap between employment structure between disabled and non-disabled persons, poor quality of life, and low economic income (5). These problems lead to poor quality of life for the visually impaired children in China. Therefore, attention should be paid to the quality of life of visually impaired children by the society, and the degree of physical, psychological, and social adaptation of disabled children should be comprehensively evaluated.

The core goal of the quality-of-life evaluation is to develop an appropriate scale. Currently, visual scales are widely used in clinical settings, and in studies in foreign countries and China, these include the 9-SF (6–8), Low Vision Quality of Life Questionnaire (LVQOL) (7), Visual Function Index-14 (VF-14) (8), Chinese version of HLVQOL (9), and Chinese version of VF-14 (10). However, these scales are used to assess the visual function of adults and are not specific for children. In recent years, a number of visual scales have been developed internationally for children. For example, the Child Visual Function Questionnaire (11) is used to measure the vision-related quality of life of children aged <7 years with visual impairment (answers by proxy), mainly to evaluate the effect of visual impairment on the personal and social behaviors of children, family, and parents, and their attitudes toward treatment. Prasad Low Vision Visual Function Scales I and II (LVP-FVQ I and II) is the first self-reported questionnaire developed in India (developing country) for children and adolescents (aged 8–18 years) to evaluate visual functional level (12, 13). However, Rasch analysis of LVP-FVQ I showed that the measurement accuracy of the scale items was lower than the acceptable level, leading to the scale's poor resolution of participants' different visual abilities and the lack of evaluation of child-related psychological characteristics. The Impact of Vision Impairment in Children (14) is used to evaluate the vision-related quality of life of children and adolescents (aged 8–18 years) with visual impairment. The scale focuses on the evaluation of daily life activities of children and adolescents. Moreover, visual-related patient-reported outcome measure has been developed rapidly in foreign countries, and there are more than 10 scales used to evaluate specific ophthalmic diseases in children (15). For example, the Child Amblyopia Treatment Questionnaire is used to evaluate the quality of life of children with amblyopia (16). In China, studies on QOL scales related to children's vision are still in their early stages. Some independently developed scales are applied to the evaluation of children's vision-related quality of life, but they are all limited to a certain eye disease and lack universality for children (17). Because of the strong cultural dependence of quality of life, foreign scales are not suitable for the Chinese context in many aspects. Thus, it is urgent to develop a more appropriate quality of life scale for children and adolescents in the Chinese context.

In light of this, this study aimed to develop a scale for children with visual impairments (Quality of Life Scale for Children with Visual Impairments [QOLS-CVI]) (see Supplementary Material), which can measure the QOL of children with disabilities in China, and evaluate the reliability and validity of the proposed scale.

## Methods

### The Development of the Quality of Life Scale for Children With Visual Impairments

#### Phase 1: Establishment of the Conceptual Framework and Items

(1) **Establishing the scale framework:** Through core group discussions, considering the particularity of the population served by the scale, the Chinese version of the Pediatric Quality of Life Inventory™ 4.0 (18) and Chinese version of the World Health Organization Quality of Life-Disability Scale for physical disability (19), which have high reliability and validity for measuring the of quality of life of children and disabled persons, respectively, were selected. Based on these well-established international scales, we determined the six domains system through adjustment and modification.

(2) **Establishment of the scale items:** The dimensions and items were sorted and analyzed by the research team, and items with the same meaning were integrated and checked for missing information to gradually form the 42 second-level items, which were screened through two rounds of the Delphi method. Moreover, 26 experts in the field of quality-of-life assessment for visually impaired children were included in this survey. The expert opinions were relatively consistent, and the results were highly reliable; after two rounds of modification, deletion, and addition, the scale comprised 41 items and 6 domains.

(3) **Scoring of the items:** All indicators were reflected against a five-point Likert scale, in which 5 represents “never,” 4 “occasionally,” 3 “sometimes,” 2 “often,” and 1 “always.” These were recorded as 5, 4, 3, 2, and 1 points, respectively. Negative items were recoded as five minus the original score (20).

#### Phase 2: The Final Scale Was Determined by Screening of the Items

In the creation of QOLS-CVI, two item selection processes and five methods of item selection were used, all of which were based on the classical test theory (CTT). The item response theory (IRT) was used as the fifth method. The IRT, also known as potential trait theory, is used to guide test preparation and selection of items. The IRT assumes that a participant has a “potential trait,” which is a concept of the participant's response to test answers (21). If an item was retained by three or more methods, we retained it. The practical significance of an item was considered before deletion. If it was meaningful, the item was kept on hold while it was sifted through a formal investigation. The following statistical analyses were performed to either retain or delete an item:

The Cronbach alpha for the total scale was 0.868, and the Cronbach alpha for the deleted items is shown in Table 1. The exclusion of one item (item number DIS-GM10) from the original 41-item scale depended on the Cronbach's alpha. If the value of the deleted items is greater than the Cronbach alpha for the total scale, then the items should be primarily adjusted (23).

**Table 1:** Comparison of demographic characteristics between visually impared and healthy children.

**Variables**	**Group**	**Visual impairments children, *n* (%)**	**Healthy children, *n* (%)**	***t*/χ^2^**	***P***
Age	X ± s	13.80 ± 2.83	14.11 ± 2.68	−1.023	0.306
Gender	Male	625 (69.8)	670 (66.07)	3.076	0.079
	Female	270 (30.2)	344 (33.93)		
Place of residence	Urban	360 (40.2)	414 (40.83)	0.072	0.788
	Rural	535 (59.8)	600 (59.17)		
Type of medical insurance	Self-paying	84 (9.4)	89 (8.8)	3.772	0.287
	Urban residents' basic medical insurance	321 (35.9)	340 (33.5)		
	New rural cooperative medical insurance	394 (44.0)	449 (44.3)		
	Others	96 (10.7)	136 (13.4)		
Grade of disability	Level 1	482 (53.9)			
	Level 2	241 (26.9)			
	Level 3	125 (14.0)			
	Level 4	47 (5.3)			
Whether to wear a visual aid or not	Yes	336 (37.5)			
	No	559 (62.5)			
Per capita household income	<1,000 RMB	359 (40.1)	123 (12.1)	1.166	0.761
	1,000–3,000 RMB	362 (40.4)	426 (22)		
	3,000–5,000 RMB	118 (13.2)	408 (40.2)		
	>5,000 RMB	56 (6.3)	57 (5.6)		
Total		895 (100)	1,014 (100)		

The corrected item-total correlation (CITC), which is the correlation of an item with the scale omitting this item, was calculated for each item, and a CITC larger than 0.20 was deemed acceptable (24).

We deleted items with factor loadings that were low (<0.4) or close to the other factors in the exploratory factor analysis (EFA) (25).

When the standard deviation (SD) of an item was ≤ 1, the corresponding item was deleted (26).

In Samejima's graded response model, the practical values of the item parameters for deletion were as follows: item discrimination parameter (a) <0.4 or difficulty parameter (b) (−3, 3) (27).

#### Phase 3: Evaluation of the QOLS-CVI

The properties of the final version of the QOLS-CVI were assessed using data from a formal investigation.

(1) **Evaluation of reliability:** Split-half reliability was used to test the consistency of the scale across its items (28). Then, test–retest reliability was conducted, which reflects the stability and consistency of a scale across time (29). The Cronbach's alpha coefficient was then used to reflect the consistency and stability of items on the scale. A Cronbach's alpha coefficient of over 0.8 generally indicates excellence, and a Cronbach's alpha coefficient between 0.6 and 0.8 indicates good internal consistency (30).

(2) **Evaluation of validity:** The relevant literature and experts were consulted in establishing content validity, which represents how well the items captured the concept of interest (31). To verify the construct validity, confirmatory factor analysis was performed to examine the structure of the QOL scale for children with visual impairments. The standardized factor loadings for an item should be > 0.5 (32). To measure the difference between the groups, a *t*-test was conducted with *P* < 0.05 set for statistical significance and in consideration of the discriminant validity of the test (32).

### Definitions

Visual impairment, also known as visual disability, refers to a certain degree of loss of visual function in individuals because of low visual acuity or visual field damage. Hence, these individuals do not achieve normal vision, thus affecting their daily life (33). The China's Federation of Persons with Disabilities defines four levels of visual disability as the standard for assessing people with disabilities. The first- and second-degree disabilities are blindness, and the third- and fourth-degree disabilities are low vision (34). The following are the International Classification of Diseases, 11th Revision [ICD-11, published by the World Health Organization (WHO)] definitions: far visual impairment, defined as visual acuity in daily life in the better of both eyes, were categporized as <0.5 (mild), <0.3 (moderate), <0.1 (severe) visual impairments, and <0.05 or central visual field <10° (blindness) (35). In 1987, China Disabled Persons' Federation formulated China's standard for visual impairment with reference to the WHO as follows: the best corrected visual acuity of the better of both eyes <0.3 with ≥0.05 and <0.05 considered as low vision and blindness, respectively. If the radius of the field of vision is <10°, no matter what the vision is, it is considered as blindness. For ease of use, we adopted the Chinese standard for visual impairment (34). The grades of visual disabilities are shown in Supplementary Table 1.

### Research Participants

A cluster sampling method was used to collect the data in this cross-sectional study from visually impaired children (and their guardians) and adolescents studying at six special educational schools in Shanxi Province and three special educational schools in Hebei Province. Ten schools in Shanxi and Hebei Provinces (five each are located in the central and suburban areas, respectively) were selected by cluster stratified sampling. Students in grades 1–6 at the primary school and grades 1–2 at the middle and high schools were selected for investigation. School leaders and teachers were consulted throughout the study to ensure minimal disruption to student learning. Data were gathered through two rounds of surveys administered by the teachers and guardians of the students, with consent from the school leaders. The study was conducted in accordance with the Declaration of Helsinki. A total of 226 children and adolescents aged over 8 years with visual impairment were included in the pre-survey. A total of 895 visually impaired children and adolescents were included in the formal investigation. Healthy students from local normal schools were selected for the survey, and 1,014 completed questionnaires were included in the analysis.

The inclusion criteria for this study were as follows: (1) visually impaired children and their guardians meeting the criteria for the diagnosis and classification of visual impairment, (2) the investigated guardians had been living with the visually impaired child for a long time (over 5 years) and had a good understanding of their physical and mental conditions and living habits, (3) participants provided consent for data collection and being able to complete the survey independently, and (4) participants who gave informed consent to participate in the evaluation voluntarily. The exclusion criteria for our study were as follows: (1) the child and their guardian were unable to complete the questionnaire even with the help of the investigators due to low educational level or other reasons, (2) patients with mental disorders and related diseases, and (3) participants whose informed consent could not be obtained.

### Procedure

Five trained investigators conducted one-on-one on-site interviews with the participants, including the reading of questions to the children and helping them choose answers, while teachers from the special training schools conducted auxiliary investigations, including distributing questionnaires on the spot, and filling in the questionnaires, which were collected through face-to-face interviews. The investigators were only responsible for reading the questions aloud, with no further explanations to avoid leading answers. After collecting the questionnaires, the collected questionnaires were strictly checked, the questionnaires with errors were eliminated, the valid questionnaires were numbered and sorted, and the EpiData version 3.1 software system (Odense, Denmark) was used for two-person dual-computer entry. The entered data was checked one by one to ensure data quality.

## Results

### Demographic Characteristics of the Visually Impaired Children

In this study, 226 visually impaired children were included in the pre-survey, and the results are shown in Supplementary Table 2. In the formal survey, 1,909 questionnaires (895 visually impaired children, 1,014 healthy children) were administered, and the baseline data of the two groups were compared using *t*-tests for continuous variables and chi-squared tests for categorical variables. Based on the results, with the significance level set at *P* <0.05, the baseline data from visually impaired children and healthy children were all comparable (Table 1).

### Formation of the Final Scale by Screening Items

**Figure 1:**
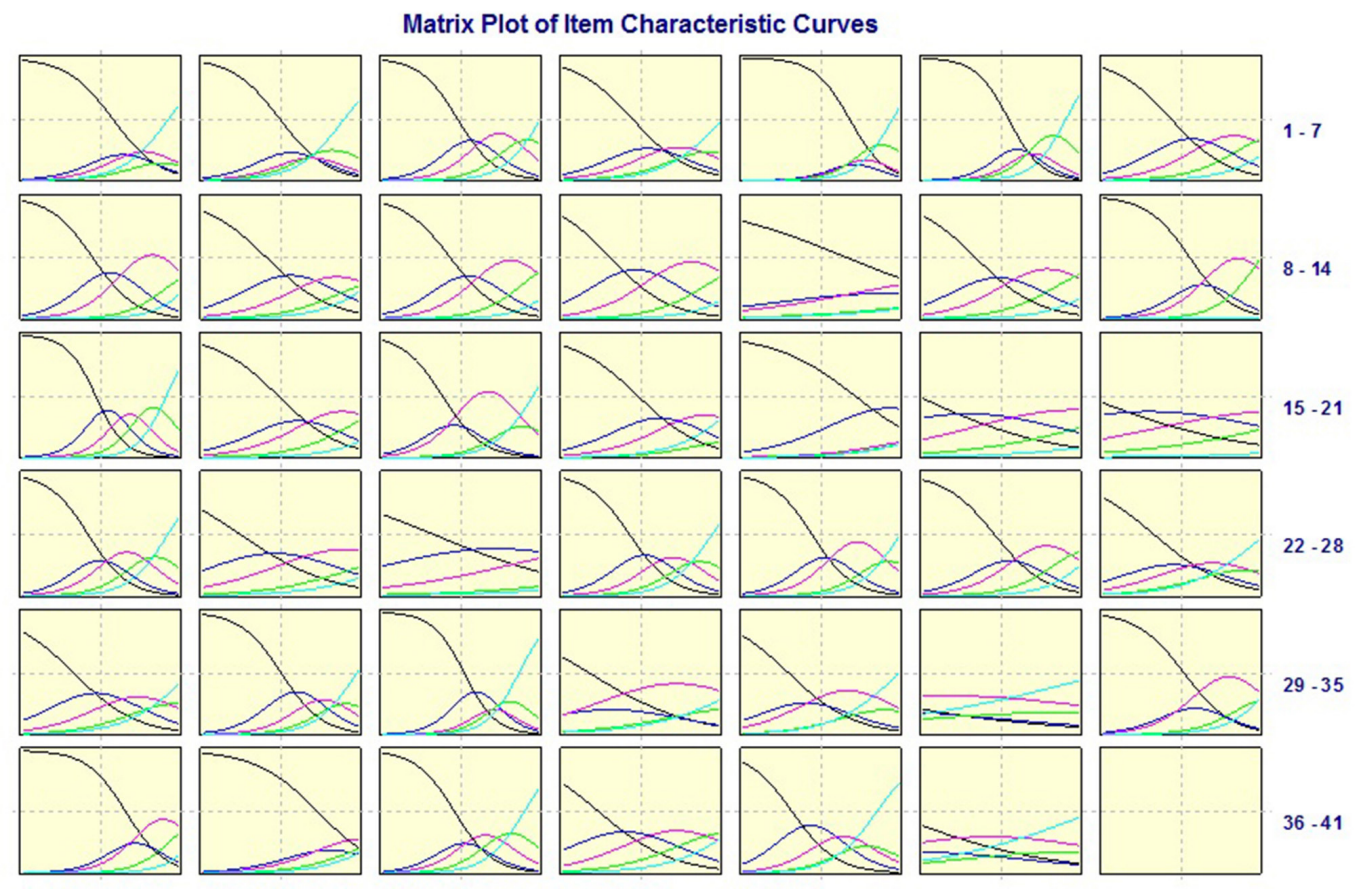
Item characteristic curve matrix diagram.

Cronbach's alpha, CITCs, SD, EFA, and IRT (Figure 1) were used to select items. According to the results shown in Table 2, three items were deleted, and the scale contained 6 domains and 38 items (see the Supplementary Material).

**Table 2:** Screening results of the second item-selection phase using CTT and IRT.

**Item**	**IRT**					**Alpha if items deleted**	**CITC**	**Factor analysis**	**SD**	**Retained**
	**a**	**b_**1**_**	**b_**2**_**	**b_**3**_**	**b_**4**_**					
PHF1	1.12	0.45	1.22	2.07	2.59	0.857	0.329	0.763	1.184	√
PHF2	1.1	−0.05	0.81	1.49	2.42	0.856	0.361	0.833	1.313	√
PHF3	1.37	−0.14	0.86	2.04	2.6	0.854	0.46	0.634	1.035	√
PHF4	0.92	−0.33	0.85	2.07	2.9	0.856	0.35	0.489	1.205	√
PHF5	1.47	1.09	1.45	1.91	2.73	0.856	0.383	0.772	**0.982**	√
PHF6	1.59	0.27	0.93	1.5	2.47	0.854	0.459	0.682	1.118	√
PHF7	0.91	−0.39	1.16	2.84	2.95	0.857	0.328	**0.361**	1.012	√
PHF8	1.21	−0.32	1	2.92	2.95	0.855	0.434	0.466	**0.904**	√
EMF1	0.82	−0.56	1.27	2.85	2.96	0.857	0.332	0.797	1.028	√
EMF2	1.09	−0.42	0.93	2.84	2.99	0.856	0.397	0.759	**0.94**	√
EMF3	0.88	−1.18	0.77	3.07	**5.02**	0.857	0.308	0.523	**0.952**	√
EMF4	**0.34**	1	3.45	7.39	**9.9**	0.861	**0.11**	0.823	1.06	√
EMF5	0.82	−0.98	0.73	2.83	2.99	0.857	0.316	0.425	1.024	√
SOF1	1.22	0.33	1.28	3.05	**8.71**	0.855	0.442	0.551	**0.857**	√
SOF2	1.8	−0.2	0.7	1.53	2.5	0.852	0.568	0.566	1.069	√
SOF3	0.82	−0.1	1.41	2.33	2.7	0.857	0.343	0.749	**0.978**	√
SOF4	1.31	−0.71	0.11	1.95	2.75	0.854	0.462	0.376	1.101	√
SOF5	0.81	−0.12	1.54	3.33	**4.01**	0.858	0.294	0.543	1.05	√
SOF6	0.65	1.31	3.95	4.89	**6.18**	0.859	0.227	0.539	**0.808**	√
ROF1	0.39	−3.19	0.65	4.85	**9.13**	0.859	0.209	0.718	1.034	√
ROF2	0.32	−3.7	1.23	6.21	**13.35**	0.86	**0.189**	0.679	**0.992**	√
ROF3	1.32	−0.49	0.42	1.55	2.56	0.853	0.487	0.602	1.189	√
ROF4	0.57	−1.55	1.01	3.83	**6.07**	0.86	**0.184**	0.754	1.056	√
ROF5	0.35	−1.03	3.7	8.46	**11.48**	0.861	**0.121**	0.783	**0.924**	×
DIS-GM1	1.23	−0.44	0.69	1.76	2.71	0.854	0.46	0.473	1.143	√
DIS-GM2	1.29	−0.35	0.65	2.12	2.88	0.854	0.464	0.639	1.057	√
DIS-GM3	1.04	−0.25	0.89	2.57	2.98	0.856	0.371	0.509	1.037	√
DIS-GM4	0.71	−1.04	0.43	1.98	2.25	0.857	0.323	0.658	1.31	√
DIS-GM5	0.83	−1.05	0.65	2.19	2.85	0.858	0.296	0.699	1.179	√
DIS-GM6	1.27	0.01	1.16	2.09	2.89	0.854	0.451	0.735	1.046	√
DIS-GM7	1.64	0.12	1.01	1.52	2.21	0.852	0.544	0.634	1.135	√
DIS-GM8	0.51	−1.98	−0.37	3.08	**4.83**	0.858	0.283	0.477	1.215	√
DIS-GM9	0.8	−1.25	0.08	1.96	2.98	0.856	0.349	0.62	1.265	√
DIS-GM10	**0.23**	−8.95	−5	0.81	**4.04**	**0.868**	−0.096	**0.377**	1.313	×
DIS-SMV1	1.11	0.11	0.9	2.76	2.82	0.856	0.396	0.632	1.013	√
DIS-SMV2	1.35	0.95	1.69	3.09	**4.32**	0.857	0.372	0.808	**0.754**	√
DIS-SMV3	0.85	1.4	2.3	3.62	**4.85**	0.859	0.228	0.847	**0.897**	√
DIS-SMV4	1.36	−0.25	0.48	1.42	2.41	0.854	0.456	0.608	1.227	√
DIS-SMV5	0.71	−1.63	0.36	2.41	2.85	0.857	0.318	0.7	1.128	√
DIS-SMV6	1.17	−1.12	0.29	1.35	2.12	0.853	0.473	0.554	1.247	√
DIS-SMV7	**0.33**	−4.42	−2.28	1.44	**3.53**	0.862	**0.136**	0.812	1.419	×

*Bold numbers represent the excluded items.*

*IRT, item response theory; CITC, corrected item-total correlation; SD, standard deviation*.

IRT was used to guide the test preparation and project selection (33). MULTILOG 7.03 software from Scientific Software International Inc. (Skokie, USA) was employed to estimate the item discrimination parameter (a) and difficulty parameter (b) of the scale. An item characteristic curve (ICC) matrix diagram was created for the items in the QOLS-CVI (Figure 1). Ideally, the blank and cyan curves should change monotonically, whereas the blue, magenta, and green curves should show a normal distribution. According to the ICC matrix, the ICCs for items 34 (DIS-GM10) and 41 (DIS-SMV7) were not ideal; therefore, they were deleted.

### Evaluation of the Final Scale (QOLS-CVI)

#### Reliability

The split-half reliability for the entire scale was 0.629. Thirty students with visual impairment at the Taiyuan School for the Blind underwent test–retest measurements 2 weeks prior to data collection, and the test–retest reliability of the total scale was 0.888. For this scale, the Cronbach alpha coefficient for the total scale was 0.841, with each domain's result shown in Table 3.

**Table 3:** Reliability of the total scale and of each domain.

**Domain**	**Split-half reliability**	**Test–retest reliability**	**Cronbach's alpha coefficient**
PHF	0.764	0.796	0.819
EMF	0.667	0.894	0.733
SOF	0.689	0.76	0.684
ROF	0.662	0.826	0.655
DIS-SMV	0.568	0.793	0.690
DIS-GM	0.513	0.801	0.701
Quality of Life Scale	0.629	0.888	0.841

*PHF, physiological function; ROF, role function; SOF, social function; EMF, emotional function; DIS-SMV, disability general module; DIS-SMV, visual disabilities specific module*.

#### Validity

**Figure 2:**
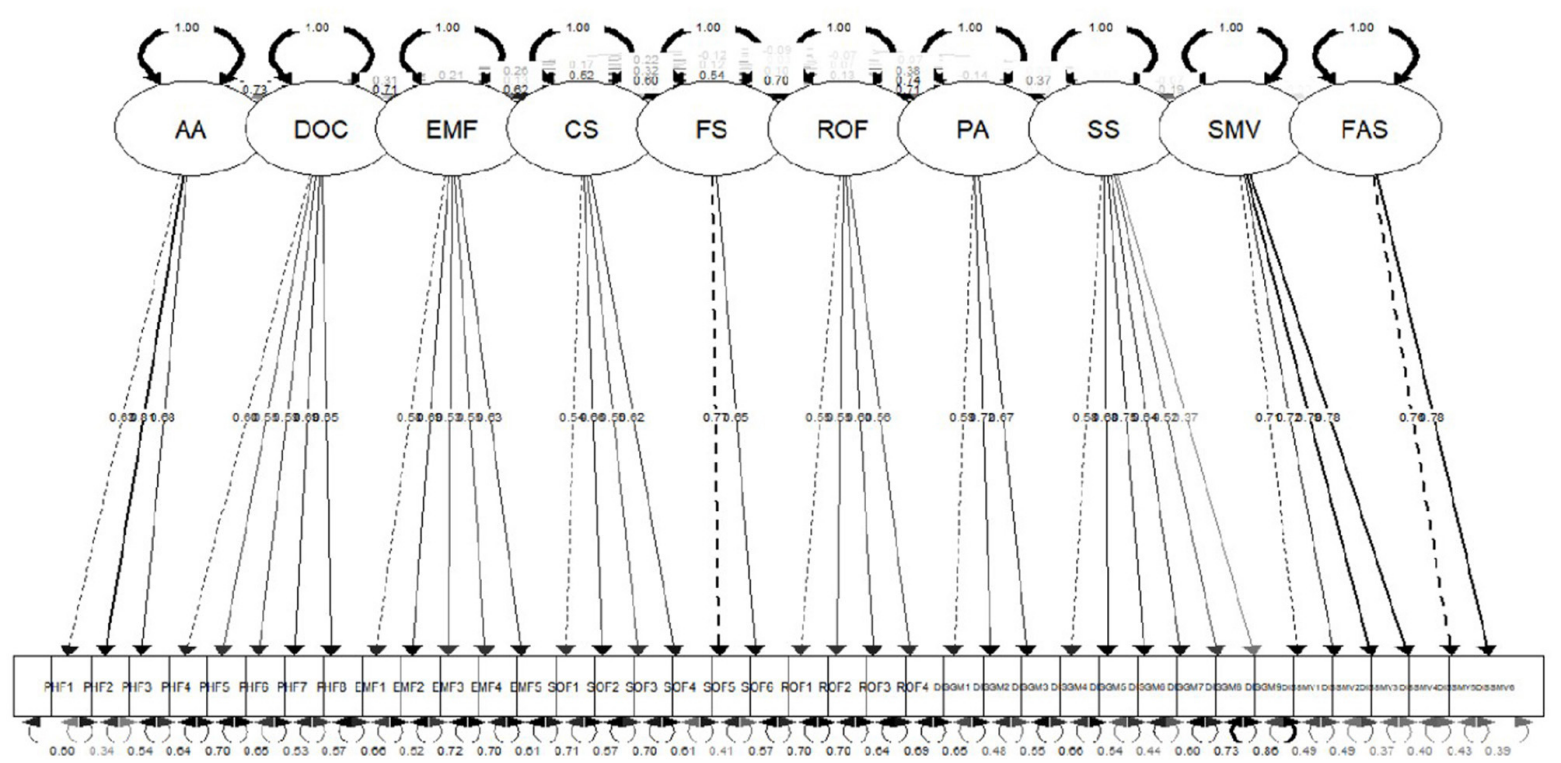
The confirmatory factor analysis of the Quality of Life Scale for Children with Visual Impairments.

(1) **Content validity:** When aiming to determine the degree of conformity between a measured subject and the prescribed content, it is impossible to determine the true value; consequently, expert evaluation is often used (36). The scale developed in this study was based on the mature, reliable, and valid QOL scales used by studies conducted in China and other countries. Concurrently, the research group consulted 26 experts in related fields as mentioned in section The Development of the Quality of Life Scale for Children With Visual Impairments, and after creating the initial items, two rounds of the Delphi method was used to screen the items. Therefore, the index system of this scale can be considered to have high content validity.

(2) **Construct validity:** Principal component analysis was used for EFA according to the characteristic root over one. The mean Kaiser–Meyer–Olkin index was 0.862. Bartlett's test of sphericity indicated that the samples were factorable at *P* < 0.001. Through maximum variance rotation, 38 items were screened and removed, and 10 subdomains were selected. The cumulative contribution rate was 57%. The structural framework of the final scale is presented in Table 4. According to the EFA results, R software (Auckland, New Zealand) was used to perform confirmatory factor analysis (CFA), and the fit indices indicated a good model fit (37) (χ^2^ = 1,848.32, df = 620, χ^2^/df = 2.981, and root mean square error of approximation = 0.048). As shown in Table 5, the model fitting indices of the scale met the corresponding requirements. The standardized factor loadings of the 10 subdomains were > 0.5. Therefore, the construct validity was deemed satisfactory, as shown in Figure 2.

**Table 4:** Scale structure of the final scale.

**Domains**	**Subdomains**	**Item**
Physiological function domain	Athletic ability	1, 2, 3
	Daily operating capacity	4, 5, 6, 7, 8
Emotional function domain	Emotional function	9, 10, 11, 12, 13
Social function	Communication skills	14, 15, 16, 17
	Family support	18, 19
Role function	Role function	20, 21, 22, 23
Disability common domain	Positive attitude	24, 25, 26
	Social support	27, 28, 29, 30, 31, 32
Visual disability specific domain	Specific module	33, 34, 35, 36
	Factor and satisfaction	37, 38

**Table 5:** Results of the confirmatory factor analysis.

**Subdomains**	**Item**	**Nonstandard factor loading**	**Standard factor loading**	**Standard error**	***t***	***P***
AA = athletic ability	PHF1	1	0.632			
	PHF2	1.542	0.81	0.092	16.758	<0.001
	PHF3	1.174	0.681	0.076	15.547	<0.001
DOC = daily operating capacity	PHF4	1	0.597			
	PHF5	0.733	0.546	0.057	12.911	<0.001
	PHF6	0.914	0.593	0.067	13.73	<0.001
	PHF7	1.088	0.686	0.072	15.178	<0.001
	PHF8	0.971	0.655	0.066	14.717	<0.001
EMF = emotional function	EMF1	1	0.579			
	EMF2	1.158	0.692	0.08	14.493	<0.001
	EMF3	0.86	0.53	0.071	12.147	<0.001
	EMF4	1.006	0.548	0.081	12.444	<0.001
	EMF5	1.088	0.625	0.08	13.629	<0.001
CS = communication skills	SOF1	1	0.543			
	SOF2	1.262	0.658	0.094	13.362	<0.001
	SOF3	1.075	0.547	0.09	11.944	<0.001
	SOF4	1.271	0.625	0.098	12.978	<0.001
FS = family support	SOF5	1	0.77			
	SOF6	0.869	0.652	0.062	13.902	<0.001
ROF = role function	ROF1	1	0.547			
	ROF2	1.005	0.552	0.084	11.955	<0.001
	ROF3	1.169	0.603	0.092	12.652	<0.001
	ROF4	0.994	0.56	0.082	12.064	<0.001
PA = positive attitude	DISGM1	1	0.594			
	DISGM2	1.138	0.721	0.078	14.619	<0.001
	DISGM3	1.223	0.669	0.087	14.091	<0.001
SS = social support	DISGM4	1	0.58			
	DISGM5	1.204	0.68	0.082	14.625	<0.001
	DISGM6	1.329	0.747	0.086	15.407	<0.001
	DISGM7	1.228	0.636	0.088	14.02	<0.001
	DISGM8	0.932	0.522	0.076	12.193	<0.001
	DISGM9	0.648	0.373	0.07	9.294	<0.001
SMV = specific module	DISSMV1	1	0.714			
	DISSMV2	1.023	0.717	0.054	18.971	<0.001
	DISSMV3	1.093	0.793	0.053	20.61	<0.001
	DISSMV4	1.073	0.776	0.053	20.272	<0.001
FAS = factor and satisfaction	DISSMV5	1		0.758		
	DISSMV6	1.142	0.368	0.783	3.106	0.002

(3) **Discriminant validity:** The discriminant validity results are presented in Table 6. The results of discriminant validity (*P* < 0.05) suggested that the QOLS-CVI was an appropriate instrument to distinguish between visually impaired children and healthy children.

**Table 6:** Score comparisons between healthy children and visually impaired children (X ± s).

**Subdomains**	**Visual impairments**	**Healthy children**	**Cohen's *d***	***t*/*t*′**	***P***
Athletic ability	5.10 ± 2.50	12.69 ± 2.94	2.78	61.05	<0.001
Daily operating capacity	9.35 ± 3.78	20.94 ± 3.80	3.07	66.69	<0.001
Emotional function	10.74 ± 3.82	20.09 ± 3.78	2.46	53.68	<0.001
Communication skills	7.97 ± 3.24	16.37 ± 3.44	2.51	54.64	<0.001
Family support	4.78 ± 2.30	7.84 ± 1.92	1.44	31.38	<0.001
Role function	8.94 ± 3.17	15.23 ± 3.40	1.91	41.60	<0.001
Positive attitude	6.64 ± 2.68	12.32 ± 2.56	2.17	47.28	<0.001
Social support	17.32 ± 5.38	18.33 ± 6.78	0.16	3.65	<0.001
Specific module	11.84 ± 4.58	16.92 ± 3.32	1.27	27.41	<0.001
Factor and satisfaction	32.90 ± 10.27	48.69 ± 9.65	1.59	34.48	<0.001

(4) **Feasibility:** The recovery rate of this scale was 100% in the pre-survey. In the formal survey, the recovery rates of the questionnaires were 99.44 and 92.18%, respectively. There were no missing items on the recovery scale, and the effective rate of the questionnaire was 100%. During the survey administration, respondents generally understood and answered the questions asked and completed the scale within 15–20 min. This shows that the content of the scale was clear, easily understood, and straightforward to complete.

## Discussion

Visual impairment is considered the sixth major disability that has caused a huge social and economic burden worldwide (38). In a person's life, visual development during childhood is particularly important. Visual impairment directly affects adults' ability to work, social skills, confidence, and family pressure (39). Therefore, it is particularly important to study the visual-related quality of life of visually impaired children in China, and provide a valid and reliable research tool to comprehensively understand the health status of visually impaired children and their ability to participate in society. This is the first study to use the method of independent research and development in constructing a QOLS-CVI in China. The QOLS-CVI comprises 6 domains, 10 subdomains, and 38 items. The results of our study indicate that the QOLS-CVI is a valid instrument for measuring quality of life among visually impaired children in China.

The QOLS-CVI analyzes the quality of life of Chinese children with visual impairment from various aspects. Compared with the existing scales for visual impairment, the QOLS-CVI has a more comprehensive assessment of quality of life (6–17). In this study, QOLS-CVI was established through discussion with experts and interviews with visually impaired children and the developed scale solved the problem of applicability and language habits among Chinese patients. The scale takes the common field of disability and the specific field of disability as independent fields, which can better evaluate the health status and rehabilitation effect of visually impaired children. Other subdomains would enhance the capacity of the QOLS-CVI to assess the effect of vision loss on the specific components of QOL. In the social field, family relationships are emphasized to recognize the importance of family support in improving the quality of life of visually impaired children.

To ensure the quality of the selection and to make the selected items more representative, independent, and sensitive, we adopted a variety of methods. In the past, the CTT method was used to select the items. Recently, IRT has become an increasingly popular method used to select items (40, 41). The IRT was used to evaluate the discrimination degree of the items, the amount of information contained in the items, and the subsequent error (42). Observation of the ICC matrix of items of the scale shows that the items contain a large amount of information, have a small amount of error, and have a high degree of discrimination. Therefore, people with different QOL can be distinguished.

Our results show that the reliability of the scale can be considered high and stable over long periods of time, and the cross index of the scale was consistent. We conducted a pre-survey among a small sample (226 visually impaired children) using a 41-item questionnaire. For the formal survey, a larger sample (895 visually impaired children and 1,014 healthy children) responded to a questionnaire with a reduced number of items (38 items) to improve the rationality of the QOLS-CVI, consistent with the requirement proposed by scholars that the sample size should be 5–10 times the number of observed variables (22). Furthermore, the overall credibility of the scale was high. Thus, the internal consistency of the scale was determined to be good, and the measurement results can be considered reliable (43). Overall, the reliability of the scale can be considered satisfactory (44, 45).

Validity is the ability of a scale to evaluate a certain ability or quality of life (46). To establish the construct validity of the QOLS-CVI, the study identified 10 subdomains according to EFA coupled with CFA, which were both independent and interrelated, and there was an inherent logical association among their items; therefore, the scale can be considered to have good structural validity (47). In the development stage of the scale, we used healthy children as a control group to evaluate discriminant validity, and the results showed that the scale could distinguish between healthy children and visually impaired children.

Previous studies have shown that blind children have fewer opportunities to practice their language skills (48). Some researchers argued that social adaptation of visually impaired children is influenced by a number of factors related to both the children's environment and the visual impairment itself (49). Consequently, we suggest that further studies and sorting of the specific module of visual disability should be conducted in the future. In summary, the overall reliability and validity of the scale is good, indicating that the model fits well.

### Limitations

Based on the initial expectations, this study investigated the quality of life of visually impaired children at different ages; however, in the actual investigation process, it was found that, first, visually impaired children lagged far behind normal children in the physical and psychological aspects; thus, their school age is evidently older; and second, early educational resources for disabled children in China are scarce. Most visually disabled children aged between 5 and 7 years do not attend kindergarten, but spend their time at home; therefore, there were almost no children aged between 5 and 7 years in this survey, and only the questionnaires of children aged above 8 years were collected.

Considering that only visually impaired children in two provinces were surveyed, the scope of the survey will need to be expanded in future studies to include more visually impaired children in more provinces and institutions, to strengthen the representativeness and applicability of the scale. Additional information about visually impaired children can be appropriately added to the scale, such as the basic information about additional disabilities, the causes of visual impairment, and type of eye disease. Utilizing the Braille questionnaire has been suggested so that visually impaired children can read and answer the questions independently, to improve the accuracy of the results; it is a convenient method to further verify the reliability, validity, and differentiation of the scale in future statistical analysis.

## Conclusion

In summary, the QOLS-CVI has good reliability and validity and can be used to accurately evaluate the QOL of visually impaired children and can be used as an evaluation tool to study the QOL and social-participation ability of children with visual impairments.

## Data Availability Statement

The raw data supporting the conclusions of this article will be made available by the authors, without undue reservation.

## Ethics Statement

This study was approved by the Institutional Review Board of Shanxi Medical University (approval number: 2018LL294). Written informed consent to participate in this study was provided by the participants' legal guardian/next of kin. Written informed consent was obtained from the individual(s), and minor(s)' legal guardian/next of kin, for the publication of any potentially identifiable images or data included in this article.

## Author Contributions

JLiu wrote the original draft, handled data curation, and performed the formal analysis. RE was responsible for the overall conceptualization, format, and language evaluation of the article. BH and CZ were mainly responsible for the overall investigation. YT, JLi, and SL participated in the data analysis. ZT served as a resource project administrator. LH and YW supervised and validated the information used in the study. Finally, JZ was mainly responsible for writing (reviewing and editing), and, in collaboration with colleagues, the study's overall conceptualization. All authors made a significant contribution to the work reported, whether that is in the conception, study design, execution, acquisition of data, analysis and interpretation, or in all these areas, took part in drafting, revising, or critically reviewing the article, provided final approval of the version to be published, have agreed on the journal to which the article has been submitted, and agreed to be accountable for all aspects of the work.

## Funding

This study was supported by National Natural Science Foundation of China (approval number 71203126).

## Conflict of Interest

The authors declare that the research was conducted in the absence of any commercial or financial relationships that could be construed as a potential conflict of interest.

## Publisher's Note

All claims expressed in this article are solely those of the authors and do not necessarily represent those of their affiliated organizations, or those of the publisher, the editors and the reviewers. Any product that may be evaluated in this article, or claim that may be made by its manufacturer, is not guaranteed or endorsed by the publisher.
