# Supplementary material for: Development and Evaluation of the Quality of Life Scale for Children With Visual Impairments in China
Source: Front Pediatr. 2022 Mar 21;10:739296. doi: 10.3389/fped.2022.739296 (PMC8979288; doi:10.3389/fped.2022.739296)
Supplement: Supplementary file 1 [file Data_Sheet_1.PDF]

NO.□□□□□

Quality of Life Scale for children with visual impairments（Above 8 years old）

To the instruction：

Hello, classmate！

This questionnaire asks questions about you and your life.Please remember what is important to you；What makes you happy?Your hopes and dreams, and your worries and concerns.

Please answer all the questions. If you are unsure of the answer to a question, choose the answer that comes closest to how you feel. This is usually your first impression.

There is no right or wrong answer, answer each question carefully.For examole， just check the corresponding number based on how much each question has bothered you in the last month：

Example					
	Aways	Occasionally	Sometimes	Often	Never
Don't you find life interesting?	1	2	3	4	5

Please tick the most suitable number according to your situation。 If you have enjoyed your life in the past two weeks, check the √ at the number 5.

Example					
	Aways	Often	Sometimes	Occasionally	Never
Don't you find life interesting?	1	2	3	4	5 √

If you need help, you can ask someone else to fill in the answers.

Your cooperation is very important for us to understand the real situation. **Thank you very much!**

★Fill in your basic information. Please tick the answer' √ '。

1.Age：_____

2.Gender：①Boy ②Girl

3.Residential area：①Urban ②rura

4.Medical insurance：①self-paying ②Urban residents' basic medical insurance ③New rural cooperative medical insurance ④Other

5.Grade of disability：①Level 1 ②Level 2 ③Level 3 ④Level 4

6.Whether to wear a visual aid or not①Yes ②No

7.Are you satisfied with the effect of your visual aid?

①Very dissatisfied ②dissatisfied ③general ④satisfied ⑤Very satisfied with

8.Per capita household income：①<1000RMB ②1000～3000RMB ③3000～5000RMB ④>5000RMB

★Check the corresponding number according to how much you have been bothered by the following questions in the last month

**About your health and activities**	☹<br>Always	Often	😐<br>Sometimes	Occasionally	☺<br>Never
1. Do I have any difficulty to walk more than 200 meters?	1	2	3	4	5
2. Do I have any difficulty to run?	1	2	3	4	5
3. Do I have trouble participating in sports and play?	1	2	3	4	5
4. Do I have trouble lifting large objects?	1	2	3	4	5
5.Do I have trouble showering by myself？	1	2	3	4	5
6.Do I have any difficulty in doing part of the housework?	1	2	3	4	5
7.Do I get hurt easily or often feel pain?	1	2	3	4	5
8.Do I feel weak？	1	2	3	4	5

About your emotional problems	☹		😐		☺
	Always	Often	Sometimes	Occasionally	Never
1.Do I feel afraid or scared？	1	2	3	4	5
2.Do I feel sad or depressed?	1	2	3	4	5
3.Do I feel angry？	1	2	3	4	5
4.Do I have trouble with sleeping?	1	2	3	4	5
5.Do I worry about something will happen to me?	1	2	3	4	5

How to get along with other children	☹		😐		☺
	Always	Often	Sometimes	Occasionally	Never
1.Did the other children say they wouldn't play with me?	1	2	3	4	5
2.Can I keep up with the other kids?	1	2	3	4	5
3.Did The other children play tricks on me?	1	2	3	4	5
4.Can't I do what other children my age can do?	1	2	3	4	5
5.Did any of my classmates help me when I was in trouble?	1	2	3	4	5
6.Can I get support and care from my parents?	1	2	3	4	5

**About your school days**	☹		😐		☺
	Always	Often	Sometimes	Occasionally	Never
1.Can't I pay attention in class?	1	2	3	4	5
2.Do I always forget things?	1	2	3	4	5
3.Can’t I keep up with my peers?	1	2	3	4	5
4.Do I never finish what I start?	1	2	3	4	5

Other problems due to physical dysfunction	☹		😐		☺
	Always	Occasionally	Sometimes	Often	Never
1.Does my current disability affect my daily life?	1	2	3	4	5
2.Will I feel inferior?	1	2	3	4	5
3.Will I feel lonely?	1	2	3	4	5
4.Am I satisfied with my ability to communicate with others?	1	2	3	4	5
5.Do I feel accepted or respected by others？	1	2	3	4	5
6.Can I get support and help from others when I meet difficulties in study or life？	1	2	3	4	5
7.Can I take care of myself？	1	2	3	4	5
8.Am I satisfied with my opportunities to participate in social activities？	1	2	3	4	5
9.Do I think my dreams and wishes will come true？	1	2	3	4	5

Other problems associated with visual impairments	☹		😐		☺
	Always	Often	Sometimes	Occasionally	Never
1.Do I think my appearance are affected？	1	2	3	4	5
2.Do I feel any change in my hearing？	1	2	3	4	5
3.Do I feel any change in my touch？	1	2	3	4	5
4.Do I feel my directional walking ability is affected？	1	2	3	4	5
5.Do I think my visual disability affects my ability to operate？	1	2	3	4	5
6.Do I think my visual impairments has a serious impact on my life	1	2	3	4	5
