# Supplementary material for: Development and Evaluation of the Quality of Life Scale for Children With Visual Impairments in China
Source: Front Pediatr. 2022 Mar 21;10:739296. doi: 10.3389/fped.2022.739296 (PMC8979288; doi:10.3389/fped.2022.739296)
Supplement: Supplementary file 2 [file Data_Sheet_2.pdf]

Supplementary Table 1. Chinese classification standards for visual impairment

Category	Grade	Good eyes have the best corrected vision
Blind	Grade 1	No light perception～<0.02, Sight radius<5°
	Grade 2	0.02～<0.05, Sight radius<10°
Low vision	Grade 3	0.05～<0.1
	Grade 4	0.1～<0.3

Note: Best corrected visual acuity refers to the best visual acuity achieved after correction with lenses.

Supplementary Table 2. Distribution of the demographic characteristics of the research participants.

General Characteristics		N	Percentage（%）
Age (years)	8-9	24	10.62
	10-11	20	8.85
	12-13	76	33.63
	14-15	41	18.14
	16-17	48	21.24
	18	17	7.52
Gender	Male	167	73.89
	Female	59	26.11
place of residence	Urban	80	35.4
	Rural	146	64.6
Type of medical insurance	Self-paying	23	10.18
	Urban residents' basic medical insurance	103	45.58
	New rural cooperative medical insurance	77	34.07
Grade of disability	Level 1	105	46.46
	Level 2	66	29.2
	Level 3	40	17.7
	Level 4	15	6.64
Whether to wear a visual aid or not	Yes	83	36.73
	No	143	63.27
Per capita household income	<1000RMB	66	29.2
	1000-3000RMB	97	42.92
	3000-5000RMB	39	17.26
	>5000RMB	24	10.62
Total		226	100
